# Migrant participation in Norwegian health care. A qualitative study using key informants

**DOI:** 10.3109/13814788.2010.525632

**Published:** 2010-11-02

**Authors:** Ursula G Småland Goth, John E Berg

**Affiliations:** 1 Oslo University Hospital—Ullevål, NAKMI, Norway; 2 Oslo University College—Faculty of Health Sciences, Norway; 3 Blakstad Hospital Vestre Viken HF, Norway

**Keywords:** Family medicine, immigrants, public health, cultural differences

## Abstract

*Background* Little is known about how migrants adapt to first-world public health systems. In Norway, patients are assigned a registered general practitioner (RGP) to provide basic care and serve as gatekeeper for other medical services. *Objectives:* To explore determinants of migrant compliance with the RGP scheme and obstacles that migrants may experience. *Methods*: Individuals in leadership positions within migrant organizations for the 13 largest migrant populations in Norway in 2008 participated in this qualitative study. Semi-structured interviews, with migrants serving as key informants, were used to elucidate possible challenges migrant patients face in navigating the local primary health-care system. Conversations were structured using an interview guide covering the range of challenges that migrant patients meet in the health-care system. *Results:* According to informants, integration into the RGP scheme and adequacy of patient-physician communication varies according to duration of stay in Norway, the patient's country of origin, the reason for migration, health literacy, intention to establish permanent residence in Norway, language proficiency, and comprehension of information received about the health system. Informants noted as obstacles: doctor-patient interaction patterns, conflicting ideas about the role of the doctor, and language and cultural differences. In addressing noted obstacles, one strategy would be to combine direct intervention by migrant associations with indirect intervention via the public-health system.

**Conclusion:**

Our results will augment the interpretation of forthcoming quantitative data on migrant integration into the public-health system and shed light on particular obstacles.

## Introduction

Migrants have changed the demographic landscape of Norway. During 2009, the number of immigrants and their children increased by more than 50 000—the largest annual increase ever recorded (1). In 2001, Norway implemented the Registered General Practitioner Scheme (RGP Scheme), in which an assigned general practitioner (assigned GP) acts as a gatekeeper to other medical services. Migrants eligible to stay in Norway for more than six months are entitled to enrol in the scheme.

According to studies in both Norway and Denmark some migrant groups report a lower rate of participation in the RGP scheme compared to natives, with a greater use of emergency clinics (2,3). Migrants might face obstacles in making use of the scheme. Unfamiliarity with a health-care system based on the RGP scheme could be one obstacle. Migrants, especially from low income or non-Western countries, may not have encountered a situation where their medical doctor holds, for instance, a gate-keeping function. Problems with doctor-patient interaction could be another obstacle. Only a few quantitative studies were found in a search of evidence-based literature exploring interaction patterns between doctors and migrant patients at GP offices in Scandinavia. Löfvander and Dyhr, as well as Brunvatne found obstacles in the doctor-patient relationship caused by communication problems and divergence in expectations about the organization of care, especially the role of the doctor as expert and the degree to which the patient is a partner in health care (4,5). Finally, Ahlberg and Duckert found that language and cultural barriers have a negative impact on treatment (6).

Bonder et al. observed that ‘Culture, individual experience and personality may affect perceptions and behaviours to self-care and health practices’ (7). In the present study, we explored determinants of migrant compliance with the RGP scheme and obstacles for participation in the RGP scheme and the Norwegian public health service that migrants may experience. With a qualitative approach, information about the com plex cultural context can be obtained and used as background knowledge for further quantitative studies.

## Methods

### General design

The study presented here, was the second part of the qualitative section of a multi-method study designed to give a comprehensive picture of migrants' participation in public health-care in Norway (8). The present qualitative study is based on semi-structured interviews with key informants from the 13 largest migrant organizations in Norway. The key informants were leaders of migrant organizations and thus represented not only themselves, but also members of their communities. The material was collected during 2008. The study was approved by the Ethical Review Board (REK Sør-øst) in Norway as project number 2008/10427.

### Qualitative study using key informants

Qualitative methods often rely on interviews of relatively few individuals with special characteristics (9). In our research, key informants were individuals chosen by their communities to serve as intermediaries because of their understanding of both the Norwegian and migrant cultures. Few members of either the migrant communities or the majority population share this expertise as culture interpreters.

By virtue of their leadership positions within their own communities, informants also have close and frequent contact with community members and are, therefore, familiar with many of the obstacles that members face. By conveying information between the minority and majority populations, key informants have the opportunity to introduce new ideas or inform about important issues. It was hoped that informants, in their role as cultural translators, would understand both perspectives and be able to explain to an observer the difficulties migrants have with the public-health system.

### Selection of key informants

Statistics Norway (SSB) estimates of the 13 largest migrant groups in Norway were used to determine countries of origin for key informants. By January 2007, in decreasing order, the largest first generation immigrant communities were (1): Poland, Sweden, Denmark, Iraq, Germany, Somalia, Pakistan, Bosnia-Herzegovina, Iran, Russia, Great Britain, Vietnam, and Turkey. Based on a contemporary political and cul tural context, we defined migrants from the European Union, North America, New Zealand, and Australia as ‘western’. Other migrants were defined as ‘non-western’.

Oslo Municipality provided a list of leaders of migrant organizations representing non-European countries, while leaders of European migrant groups, who do not collaborate with Oslo municipality, were referred by their respective embassies.

Since informants in the study served as cultural mediators/cultural translators, it was critical that they were well-integrated into Norwegian society, fluent in Norwegian and familiar with the systems and values of the majority population (7,9). Finally, it was required they had intimate knowledge of their own group and culture.

The project leader, a migrant herself, contacted the informants, all of whom had personally migrated to Norway. It is noteworthy that migrants from the 13 ethnic minority backgrounds were not a homogeneous group with respect to language abilities or length of residence.

### Interviewing and recording

The 13 interviews were conducted by the project leader in Norway at sites chosen by the informants. Interviews lasted between 60 and 90 min. Semi-structured questions based on existing literature focused on informants' opinions about experiences of members of their communities within the RGP scheme (2,4–7,9). Areas **s**uch as doctor-patient communication, language difficulties, equal provision of health services, and adequacy of information provided about the public-health system were discussed. No recording device was used to create an environment of trust and openness. Notes were taken during and immediately following the interviews. Respondents were assured anonymity and gave informed consent.

To improve accuracy and internal validity, we conducted a pilot interview of a key informant for the Latin America community (Chile, Columbia and Peru) and performed a ‘member check’, i.e. the interviewer showed the notes of the interview to the participant to find out whether the notes correctly represented her ideas.

### Analysis

The qualitative data collected for this study do not permit scientifically-based generalizations. To reduce potential bias and to assess the consistency of the information obtained from the informants, the information was validated by a co-researcher. After the interview, both the researcher and a co-researcher read the interview notes individually. They independently made their interpretations. These individual results were compared and discussed before a final conclusion was drawn.

## Results

We sorted results of the interviews under the following seven headings.

### Health-related challenges

Migrant informants noted that mental-health related problems, often found in migrants who are refugees or have sought asylum, are caused by present or past conflicts. This leads, in their opinion, to alcohol abuse, which is recognized and treated. What is not treated, in their opinion, is the cause—the conflicts and traumas of the present and past. Based on their knowledge of specific individuals, informants explained that such individuals, because of experiences in their home countries, give only partial information to each of several doctors, both in an attempt to hide their alcohol problems and because of a reluctance to confide all information about themselves to a single individual. Informants noted that substance abuse also could be found among migrants who have experienced trauma.

According to informants, migrants with longer residence in Norway are more likely to develop lifestyle-related diseases, such as obesity, diabetes II, and cardiovascular disease. Labour migrants, engaged in blue-collar work, often present with musculoskeletal diseases.

### Factors enhancing participation

Migrant informants stated that, according to their experience, participation of their members in the RGP scheme depended on both the existence of a similar system in their former home country, length of stay in Norway, and intention to settle in Norway.

Informants noted that the Norwegian Labour and Welfare Organization (NAV) sends routine information about the RGP scheme only in Norwegian, which leads to scanty or incorrect knowledge about the system among migrants (especially among labour- and family-reunification migrants), who may have insufficient knowledge of Norwegian. Some informants mentioned that a generally low level of medical literacy could also pose difficulties for understanding the system. Informants from Pakistan, Poland, and Vietnam reported that older women often make decisions for their entire families about when to see the doctor.

Based on interviews with migrant-association informants, challenges migrants may experience with the Norwegian health-care system and the RGP scheme seem to be inversely related to their competence in Norwegian and intention to stay in Norway, and vary with the cause for migration.

### Experiences within the RGP scheme

Migrant experiences with the RGP scheme varied considerably. Migrants from countries geographically close to Norway were satisfied with the RGP scheme. The major complaint was long waiting times before an appointment. All informants expressed the opinion that experiences with the RGP scheme are positively related to length of residence. The informants assumed that this was correlated with an increased understanding of the system and the role of the assigned GP.

### Experience with the assigned GP

Migrant informants articulated the positive experiences that members of their organizations have had with their assigned GP, but migrants from non-European countries, as well as migrants from Poland often perceived their GP as unprofessional or not well-educated. A frequent reason cited was the doctor's recourse during office visits to reference literature, such as the Norwegian Pharmaceutical Product Compendium (Felleskatalogen), which patients viewed as a sign of ignorance. Another complaint was that doctors failed to understand the importance of pain as signifying a medical problem.

Some informants said that the satisfaction with the individual GP was related to language skills—the better the understanding by the doctor, the better the satisfaction of the patient. However, the use of public translation services (whose availability is mandated by law) also reportedly led to problems. Informants commented that the use of a translator cuts into the consultation time available at the GP's office. If a translator comes from a migrant's own community, patients also worried about their professional conduct for maintaining confidentiality. Translators, compatriots believe, have been the source of rumours. Fear of using a public translator with a migrant background may be problematic for some refugees and asylum-seekers. As a result, family members or close friends are often preferred as translators.

According to some informants, some of their members believe that in acting as a gatekeeper the GP fails to refer migrants to specialized services as desired, so that patients' expectations about treatment are unfulfilled.

The attitude and abilities of the doctor were experienced as confusing and different from their home system. Interaction patterns or role understanding for both doctors and patients differed in the informants' eyes from expectations and may lead to insecurities. This was mainly elaborated by non-Western migrant informants.

### Gender specific challenges with the assigned GP

Most female immigrants of non-western origin will not consent to a male assigned GP for a gynaecological examination, or even be alone with a male assigned GP in a closed room. At the same time the patient is often not aware of the possibility of changing assigned GPs, and requesting a female doctor.

### Equal treatment

Migrant informants expressed different perceptions regarding whether migrants receive similar levels of care as Norwegians. Migrants from Somalia, Pakistan, Poland, Turkey and Russia expressed that many migrants got a lower level of treatment.

### Emergency clinic versus assigned GP

Informants suggested that the frequency of and reasons for use of emergency wards vary according to the migrant's country of origin, age, and reason for migration. They cited ease of accessibility, familiarity with the home system and what migrant informants perceived as a lack of information about and/or accessibility to their GP as reasons for preferring emergency clinics to GP appointments. Use of emergency clinics by labour migrants with a contract of less than 6 months can be explained by the fact that they are not legally entitled to use an assigned GP.

Informants also reported that patients who did not receive the desired medical treatment from their assigned GP had a tendency to seek help at the emergency clinic. Informants from countries in conflict mentioned that some of their members (i.e. refugees and asylum seekers) fear that relying on one doctor would mean that the doctor would know everything about them, and that this information could later be used against the migrant.

## Discussion

### Main results

In this paper, we examined opinions about the registered GP scheme in 13 different migrant groups in Norway as expressed by key informants. The main points mentioned in this study are differences in understanding of disease between doctors and patients, surprise at the role of the Norwegian GPs, insufficient understanding of information about the RGP scheme and a sense of receiving a lower standard of care among non-European informants. Overcoming language barriers appears to be a significant factor in increasing migrants' access to the GP scheme.

### Equal access to care

Equal access to health care for those in need is basic to achieving equity in health, regardless of personal finances (10,11). Norway has a tax-financed health care system in which patients pay nothing for hospital treatment, and are responsible for only a very small co-payment for basic care, making direct financial outlays for health care close to zero. Although most migrants in Norway have the same legal access as ethnic Norwegians to public health services, this study indicates that many barriers to obtaining equal access to health-care still exist as mentioned by the key informants.

### Lack of shared understanding

An earlier study by Ahlberg and Duckert, corroborate our findings that minority patients and ethnic Norwegians have a different understanding of disease (6). They underscore the importance of adapting treatment institutions and GP offices to the changing demographics of the population. In a recent paper, Sudore et al. found that a lack of shared proficiency in any language was a more significant obstacle than lack of health-literacy in doctor-patient interactions involving migrants (12).

### Information challenges

Comments by key informants from non-western countries concerning treatment standards may reflect problems stemming from lack of language proficiency. Additionally, studies have found that language proficiency, cultural views, and age were more important in patient's evaluation of care than ethnic origin or education (13,14). In the present study, participation in the GP Scheme was more likely, as indicated by the key informants, when migrants were aware of information about the system and were able to gain an understanding of how it functions. Language barriers were shown to contribute to a lack of understanding of the system and a lack of confidence in the GP, resulting in greater use of emergency treatment options (15). The key informants indicated that when information about the scheme was not understood, individuals often failed to take actions that could help them benefit more fully from the system. This may include changing to a female doctor for some female patients, or choosing a new GP after moving to a new location. If the patient needs are not met, previous studies have shown that overuse of emergency services may result (3,16,17). Misunderstanding of communication attempts between doctor and patient seems to be a general finding as shown in both a Norwegian and German study (14,15).

### Patient satisfaction

Lurås found, in a study with a representative sample of 2 326 patients who had visited their GPs within the last six months, that a reduced patient list was correlated with higher patient satisfaction (18). The result was unrelated to patient ethnicity. Whether her finding could explain some of the dissatisfaction with the GP found in the present study cannot be determined from our qualitative analysis. Mjaaland & Finset showed that Norwegian GPs are not using resource and coping strategies, a set of strategic activities, to achieve equity in their daily work and that only 2% of utterances in videotaped consultations could be categorized as resource or coping-oriented (19). This may very well be more important for migrant patients.

### Strengths and limitations

The article is not a general statement of the situation of all migrants from a given country, but the expressed opinion of the key informants. The present study has focused on the 13 largest communities of migrants. There is, however, a large degree of diversity, both within the survey group and among all migrants in Norway, which limits the generalizability of our findings. However, such diversity sheds light on the very different needs of each migrant group.

Data collection performed without recording or a note-taking partner during the interview limits outcome validation but may augment the value of the information received.

This study has focused on experiences with GPs in the Scheme and not with those in an unregulated setting. The GP Scheme as described here is specific to Norway. Comparisons with the Norwegian system might also be difficult because of organizational and financial peculiarities. However, findings regarding the interaction of migrants with the local general practitioner system could be relevant for other European countries.

### Implications

The lack of understanding of disease, a different expectation regarding the role of the doctor and the lack of received general information on the RGP scheme represent, in our opinion, the main points for further study.

Use of GPs compared to use of emergency services, the diagnoses registered and indirect measures of standard of care given will be further scrutinized in a forthcoming quantitative study of consultation data for all citizens in Norway.

The challenges discussed in the present study may be overcome by systematically distributing information through existing channels, such as migrant organizations, and by providing communication training and awareness programmes for GPs to meet migrant needs better (20).

## Conclusion

The intention of the study was to determine possible major challenges towards the existing primary health care system. How patients experience the primary health care service is what really matters. Equity in health-care consists of more than cultural competency as a technical skill; it also includes interpersonal skills and the adaptation of the RGP system.
